# Adjuvant chemotherapy improves survival of patients with high-risk upper urinary tract urothelial carcinoma: a propensity score-matched analysis

**DOI:** 10.1186/s12894-017-0305-4

**Published:** 2017-12-01

**Authors:** Kazutoshi Fujita, Kei Taneishi, Teruo Inamoto, Yu Ishizuya, Shingo Takada, Masao Tsujihata, Go Tanigawa, Noriko Minato, Shigeaki Nakazawa, Tsuyoshi Takada, Toshichika Iwanishi, Motohide Uemura, Yasushi Okuno, Haruhito Azuma, Nonomura Norio

**Affiliations:** 1 Department of Urology, Osaka University Graduate School of Medicine, 2-2 Yamada-oka, Suita, Osaka, 565-0871 Japan; 2 Department of Clinical System Onco-Informatics, Graduate School of Medicine, Kyoto University, Kyoto, Japan; 3 Department of Urology, Osaka Medical College, Takatsuki, Osaka, Japan; 4 Department of Urology, Osaka Medical Center for Cancer and Cardiovascular Diseases, Osaka, Japan; 5 Department of Urology, Osaka General Medical Center, Osaka, Japan; 6 Department of Urology, Osaka Police Hospital, Osaka, Japan; 7 Department of Urology, Sumitomo Hospital, Osaka, Japan; 8 Department of Urology, Osaka Rosai Hospital, Sakai, Osaka, Japan; 9 Department of Urology, Nishinomiya Prefectural Hospital, Nishinomiya, Japan; 10 Department of Urology, Minoh Municipal Hospital, Minoh, Japan; 11 Department of Urology, Higashi Osaka General Medical Center, Higashi-, Osaka, Japan

**Keywords:** Upper urinary tract urothelial carcinoma, Adjuvant chemotherapy, Sodium, Hemoglobin

## Abstract

**Background:**

The purposes of this study were to determine whether adjuvant chemotherapy (AC) improved the prognosis of patients with high-risk upper urinary tract urothelial carcinoma (UTUC)and to identify the patients who benefited from AC.

**Methods:**

Among a multi-center database of 1014 patients who underwent RNU for UTUC, 344 patients with ≥ pT3 or the presence of lymphovascular invasion (LVI) were included. Cancer-specific survival (CSS) estimates were calculated by the Kaplan-Meier method, and groups were compared by the log-rank test. Each patient’s probability of receiving AC depending on the covariates in each group was estimated by logistic regression models. Propensity score matching was used to adjust the confounding factors for selecting patients for AC, and log-rank tests were applied to these propensity score-matched cohorts. Cox proportional hazards regression modeling was used to identify the variables with significant interaction with AC. Variables included age, pT category, LVI, tumor grade, ECOG performance status and low sodium or hemoglobin score, which we reported to be a prognostic factor of UTUC.

**Results:**

Of the 344 patients, 241 (70%) had received RNU only and 103 (30%) had received RNU+AC. The median follow-up period was 32 (range 1–184) months. Overall, AC did not improve CSS (*P* = 0.12). After propensity score matching, the 5-year CSS was 69.0% in patients with RNU+AC versus 58.9% in patients with RNU alone (*P* = 0.030). Subgroup analyses of survival were performed to identify the patients who benefitted from AC. Subgroups of patients with low preoperative serum sodium (≤ 140 mEq/ml) or hemoglobin levels below the normal limit benefitted from AC (HR 0.34, 95% CI 0.15–0.61, *P* = 0.001). In the subgroup of patients with normal sodium and normal hemoglobin levels, 5-year CSS was 77.7% in patients with RNU+AC versus 80.2% in patients with RNU alone (*P* = 0.84). In contrast, in the subgroup of patients with low sodium or low hemoglobin levels, 5-year CSS was 71.0% in patients with RNU+AC versus 38.5% in patients with RNU alone (*P* < 0.001).

**Conclusions:**

High-risk UTUC patients, especially subgroups of patients with lower sodium and hemoglobin levels, could benefit from AC after RNU.

## Background

Localized upper urinary tract urothelial carcinoma (UTUC) is treated by radical nephroureterectomy with bladder cuff incision (RNU). However, approximately 30% of patients with localized UTUC suffer disease recurrence and have poor survivals [1]. To improve the prognosis, perioperative chemotherapy before or after surgery was performed. Because of the problem of losing renal function after RNU, neoadjuvant chemotherapy may be better for the patients with high-risk UTUC. However, it is difficult to predict UTUC with adverse pathology preoperatively.

Postoperatively, patients with adverse pathology can be selected for adjuvant chemotherapy (AC), and the overtreatment of the patients with low-risk UTUC can be prevented. In contrast, patients who undergo RNU suffer the loss of renal function resulting in their ineligibility for chemotherapy.

There are limited reports of AC for UTUC patients, but the efficacy of AC for UTUC patients remains controversial [2–7]. No prospective randomized trials have investigated the efficacy of AC for UTUC.

Previously, we reported that lower levels of serum sodium (Na < 141 mEq/L) and hemoglobin (lower than normal range) could predict the prognosis of patients with UTUC who underwent RNU. The subset of patients with high-risk UTUC (≥ pT3, presence of lymphovascular invasion [LVI], or positive lymph nodes) could have a good prognosis and might not benefit from AC to improve survival.

Therefore, the primary purpose of this study was to investigate the effect of AC for high-risk UTUC patients who underwent RNU, and the secondary purpose was to seek effective predictors of AC to select the patients who could benefit from its use.

## Methods

### Patients

We used a database including 1014 patients with UTUC who underwent RNU between 1998 and 2013 at Osaka University Hospital, Osaka Medical College Hospital, and their affiliated hospitals. Among these patients, 359 with localized high-risk UTUC (≥ pT3 or LVI positive and pN negative) were identified. Five patients received neoadjuvant chemotherapy, and 2 patients with incomplete resection were excluded. Eight patients who received only 1 cycle of AC due to side effects were also excluded. Thus, we retrospectively analyzed the remaining 344 patients. RNU was performed laparoscopically in 188 patients (54.7%) and by laparotomy in 156 patients (45.3%). Lymph node dissections were performed in an extended or limited manner, at the surgeon’s discretion. The following clinical and pathological data were obtained from the database: age; sex; Eastern Cooperative Oncology Group (ECOG) performance status (PS); pathological tumor, lymph node, metastasis (TNM) classification; presence of LVI; tumor grade; tumor lesion location; and follow-up data. Serum sodium and hemoglobin levels were measured less than 1 month before RNU. Patients were followed-up every 3 months during 0–2 years after surgery, every 6 months during 2–5 years, and every 6–12 months thereafter. Tumor recurrence was defined as the development of local recurrence, distant metastasis, and/or lymph node metastasis; tumor recurrence did not include intravesical recurrence. Follow-up examinations consisted of routine blood test, urine cytology, cystoscopy, and the chest and abdominal computed tomography scans. This study was approved by the Institutional Review Board of Osaka University Hospital.

### Statistical analysis

Clinical characteristics were analyzed using the Mann-Whitney U test and Fisher’s exact test. The association between AC and patient cancer-specific survival (CSS) were tested by Kaplan-Meier survival curve analysis and log-rank tests. Propensity score matching was used to adjust the confounding factors for selecting patients for AC. A logistic regression model, which included age, sex, ECOG PS, pathological findings (pT stage, LVI status, tumor grade), was used to estimate each patient’s probability of receiving AC. Patients with RNU only were matched on a one-to-one basis with patients with RNU + AC based on nearest-neighbor matching. To assess the factors affecting CSS, a Cox proportional hazard model was used. Variables included age, pT category, LVI, tumor grade, ECOG PS, and low sodium or hemoglobin score, which we previously reported to be a prognostic factor of UTUC [1, 8]. All of statistical tests were performed with SPSS version 11.0 (SPSS, Chicago, IL, USA) and GraphPad Prism 5 (GraphPad Software, La Jolla, CA, USA). Probability values (*P*) were two-sided, and statistical significance was defined as a *P* < 0.05.

## Results

### Analysis in the overall cohort

**Fig. 1:**
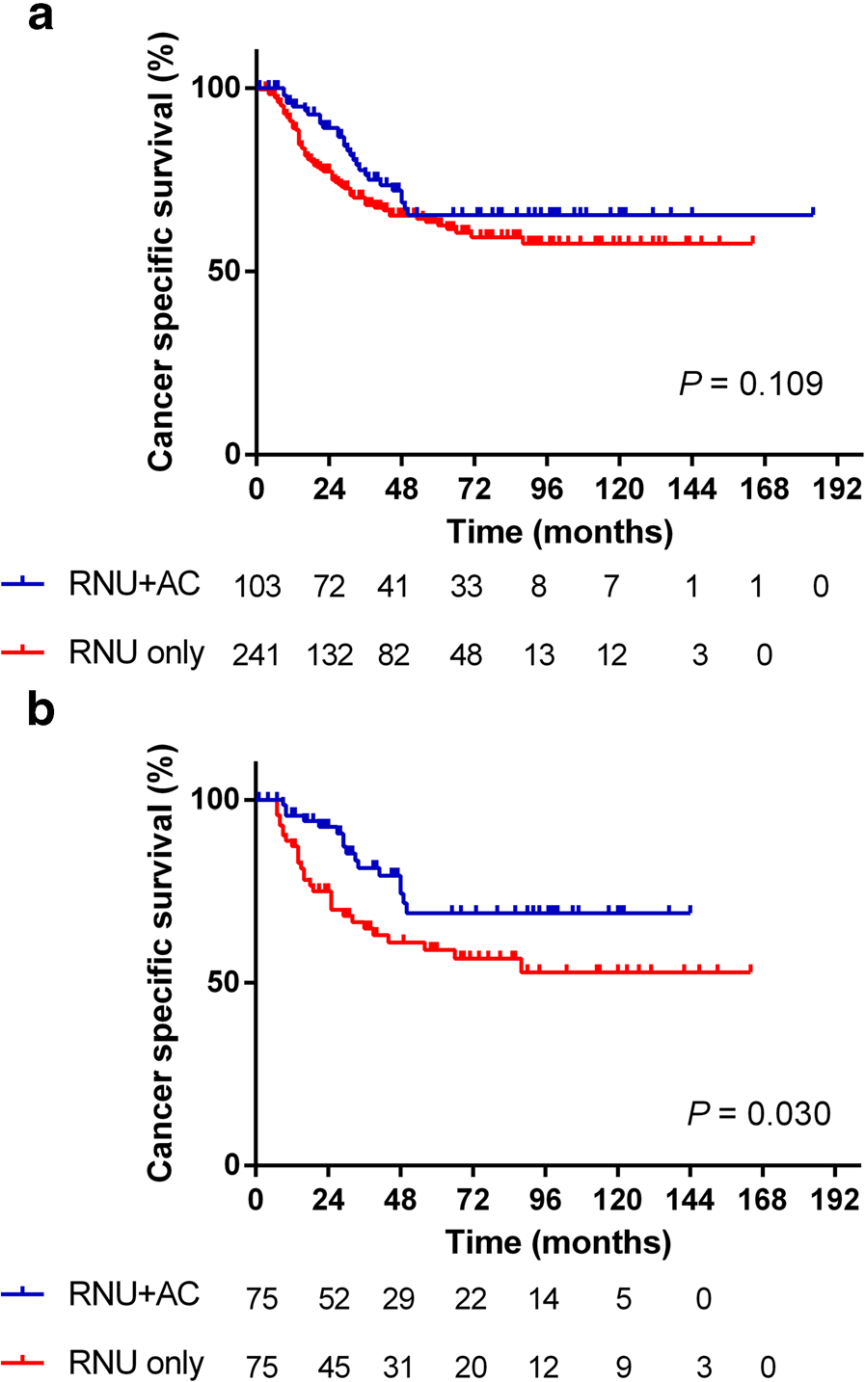
Cancer-specific survival of the overall cohort (**a**) and the propensity score-matched cohort (**b**) (solid line: patients with RNU + AC, dashed line: patients with RNU alone). AC, adjuvant chemotherapy; RNU, radical nephroureterectomy

Among the 344 high-risk patients, 103 (29.9%) patients received AC. A median of 2 cycles (range 2–4 cycles) of platinum-based AC were administered. Patient characteristics are summarized in Table 1. There were several factors that differed significantly between the patients with RNU alone and those with RNU + AC. The median follow-up was 32 months (range 1–184 months), with overall 2- and 5-year CSS of 80.7% (95% CI 75.7–84.7%) and 63.1% (95% CI 56.6–68.8%), respectively. The Kaplan-Meyer curve for the overall cohort showed that no significant differences were found in overall survival between the patients with RNU alone and those with RNU + AC (log-rank test, *P* = 0.109) (Fig. 1a).

**Table 1:** Patient characteristics of overall cohort (*n* = 344)

	RNU only	RNU plus AC	*P* value
n (%)	241 (70)	103 (30)	
Age (years)(median (range))	74 (34–91)	66 (28–82)	< 0.0001
Gender, n (%)			0.20
Male	166 (69)	78 (76)	
Female	75 (31)	25 (24)	
ECOG performance status			0.40
0–1	199 (83)	91 (88)	
2–4	13 (5)	3 (3)	
Unknown	29 (12)	9 (9)	
Pathological T stage, n (%)			0.007
≤ T2	70 (29)	18 (17)	
T3	158 (66)	84 (82)	
T4	13 (5)	1 (1)	
Tumor grade, n (%)			0.056
G1	13 (5)	3 (3)	
G2	90 (37)	27 (26)	
G3	138 (58)	73 (71)	
LVI, n (%)			0.003
Absent	107 (44)	29 (28)	
Present	128 (53)	73 (71)	
Unknown	6 (3)	1 (1)

### Propensity score-matched analysis

Because selection bias for AC would exist, we matched the patients using propensity scores for the use of AC, resulting in matched cohorts of 75 patients with RNU only and 75 patients with RNU + AC. The propensity score-matched cohorts are summarized in Table 2. The differences in the variables between the two groups decreased after propensity score matching. In the propensity score-matched cohort, patients with RNU + AC had a better survival rate significantly than the patients with RNU only (Fig. 1b). The 2- and 5-year CSS were 92.6% (95% CI 83.3–96.8%) and 69.0% (95% CI 53.8–80.1%) for patients with RNU + AC compared with 75.0% (95% CI 62.8–83.7%) and 58.9% (95% CI 45.5–70.1%), respectively, for patients with RNU only (HR 0.51, 95% CI 0.28–0.93; *P* = 0.030).

**Table 2:** Patient characteristics of propensity score matched cohort (*n* = 150)

	RNU only	RNU plus AC	*P* value
n	75	75	
Age (years)(median (range))	66 (34–85)	68 (28–82)	0.62
Gender, n (%)			0.28
Male	50 (67)	56 (75)	
Female	25 (33)	19 (25)	
ECOG performance status, n (%)			1.0
0–1	72 (96)	72 (96)	
2–4	3 (4)	3 (4)	
Pathological T stage, n (%)			0.51
≤ T2	12 (16)	15 (20)	
T3	60 (80)	59 (79)	
T4	3 (4)	1 (1)	
Tumor grade, n (%)			0.84
G1	2 (2)	1 (1)	
G2	23 (31)	23(31)	
G3	50(67)	51 (68)	
LVI, n (%)			1.0
Absent	24 (32)	23 (31)	
Present	51 (68)	52 (69)	

### Subgroup analysis to identify the predictive marker for AC

**Fig. 2:**
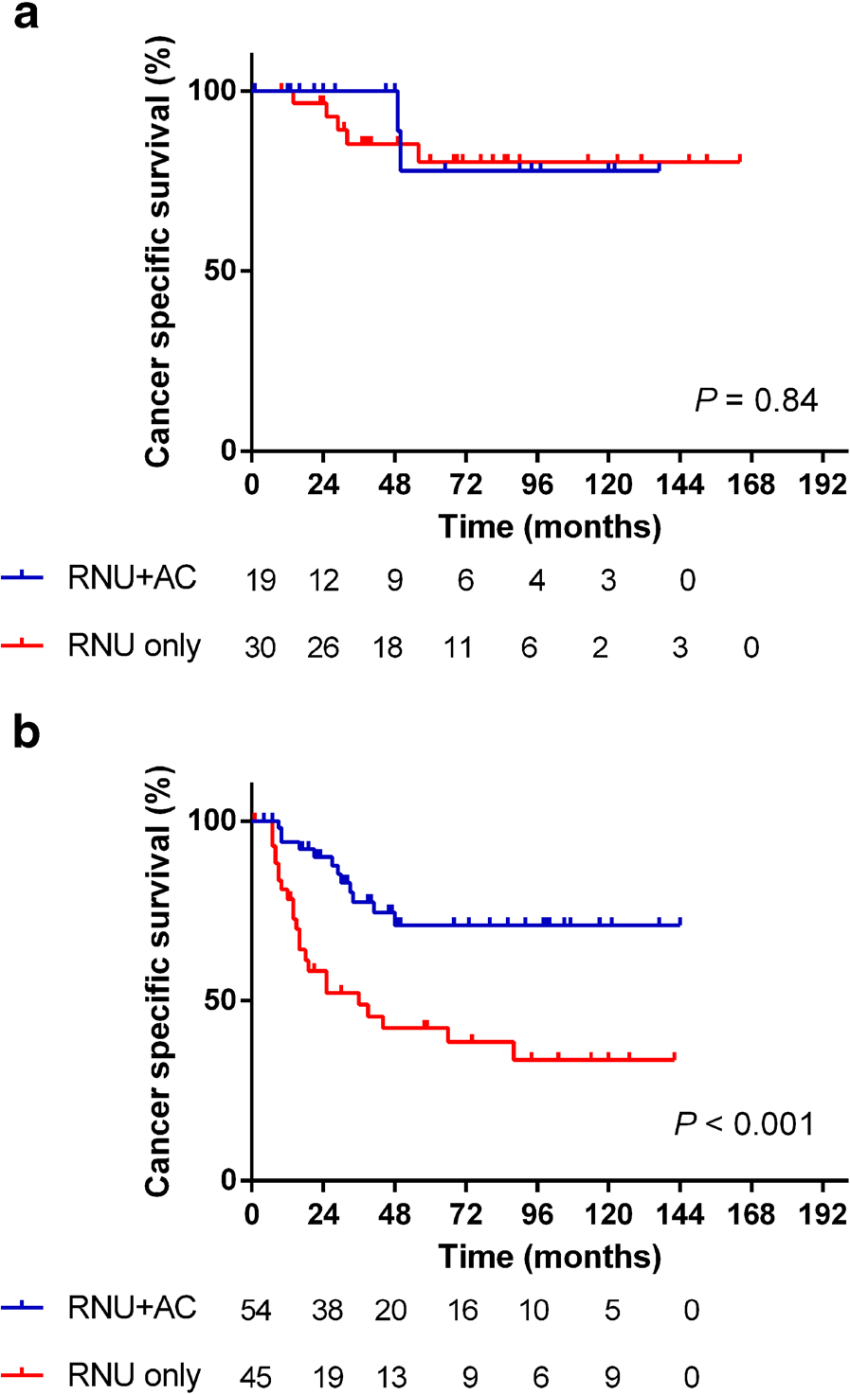
Cancer-specific survival of propensity score-matched cohort stratified by preoperative sodium and hemoglobin levels. **a** Patients with normal sodium and normal hemoglobin levels. **b** Patients with low sodium or low hemoglobin levels. (solid line: patients with RNU + AC, dashed line: patients with RNU alone). AC, adjuvant chemotherapy; RNU, radical nephroureterectomy

Subgroup analyses of survival were performed to identify the patients who benefitted from AC to improve CSS. Subgroups of patients with low preoperative serum sodium (≤ 140 mEq/ml) or hemoglobin levels below the normal limit, the presence of LVI, or tumor grade 3 had received benefits from AC (HR 0.34, 95% CI 0.15–0.61, *P* = 0.001; HR 0.51, 95% CI 0.26–0.98, *P* = 0.046; HR 0.41, 95% CI 0.21–0.81, *P* = 0.011, respectively) (Table 3). AC for the patients with these factors resulted in improved survival. In patients with normal sodium and normal hemoglobin levels, the 5-year CSS was 77.7% in the patients with RNU + AC versus 80.2% in the patients with RNU alone (log rank test, *P* = 0.84). In contrast, in the patients with low sodium or low hemoglobin levels, the 5-year CSS was 71.0% in the patients with RNU + AC versus 38.5% in the patients with RNU alone, resulting in a 32.5% improvement in 5-year CSS (log rank test, *P* < 0.001) (Fig. 2). These results would suggest that the patients with normal sodium and hemoglobin levels would have good prognosis and would not need to receive AC.

**Table 3:** Subgroup analysis to identify the patients who benefit from adjuvant chemotherapy

	RNU only	RNU + AC	HR	95% CI	*P* value
age					
≤ 70	14/48	7/49	0.46	0.18–1.1	0.099
> 70	14/27	9/26	0.56	0.24–1.3	0.19
pT2					
≤ 2	4/12	4/15	0.66	0.16–2.6	0.56
3	22/60	12/59	0.51	0.25–1.0	0.066
4	2/3	0/1	–	–	1
Na-Hb score					
0	5/30	2/19	0.84	0.16–4.3	0.84
1–2	23/45	12/54	0.30	0.15–0.61	0.001
ECOG PS					
0–1	27/72	16/72	0.542	0.292–1.01	0.053
≥ 2	1/3	0/3	–	–	1
LVI					
–	4/24	2/23	0.483	0.088–2.64	0.40
+	24/51	14/52	0.51	0.26–0.98	0.046
Grade					
1–2	3/25	3/24	0.99	0.20–4.9	0.99
3	25/50	13/51	0.41	0.21–0.81	0.011

## Discussion

UTUC is a rare disease with poor prognosis. More than 40% of patients have advanced-stage cancer at diagnosis, and their prognosis is poor [1]. To improve survival, perioperative chemotherapy is performed. The efficacy of neoadjuvant chemotherapy (NAC) for urinary bladder cancer had been confirmed by randomized study. Immediate AC for patients with advanced urinary bladder cancer did not improved overall survival over that of patients who underwent deferred chemotherapy, but it might benefit a subgroup of urinary bladder cancer patients, especially pN-positive patients [9]. After RNU, many patients lose nearly 50% of their renal function and can be ineligible to receive chemotherapy [10]. From these points of view, NAC might be preferred for the patients with advanced UTUC. However, the precise preoperative diagnosis of tumor stage or LVI status is difficult, although one study showed the usefulness of magnetic resonance imaging for the prediction of tumor stage [11]. Unlike urinary bladder cancer, for which pathological stage can be accurately diagnosed by transurethral resection of the bladder tumor before radical cystectomy, the accurate staging of UTUC is difficult even with a ureteroscopic biopsy [12].

Because UTUC is a rare malignancy comprising 5% of all urothelial cancer, it is difficult to enroll enough UTUC patients to adequately perform a prospective, randomized study to prove the efficacy of perioperative chemotherapy. For lymph node-positive UTUC patients, the efficacies of adjuvant chemotherapy were reported. Retrospective analysis of 74 lymph node-positive UTUC patients showed the AC improved CSS compared with RNU alone (HR 0.52, 95%CI 0.24–0.82, *P* = 0.014) [2]. Retrospective analysis of 263 lymph node-positive UTUC patients showed that AC did not improve CSS in overall patients (HR 0.89, *P* = 0.49), but improved CSS in the subgroup of patients with pT3–4 N+ (HR 0.67, *P* = 0.022) [13]. Retrospective analysis of 109 locally advanced UTUC patients (pT3–4pN0/xM0) showed that cisplatin-based AC improved recurrence-free survival (HR = 0.41, *P* = 0.017) and CSS (HR 0.33, *P* = 0.037) [14]. Propensity-matched analysis of 1544 UTUC patients with pT2-4 N0 or lymph node-positive showed that AC did not improve overall survival compared with RNU alone (HR 1.14, 95%CI 0.91–1.43, *P* = 0.268). The largest study recently reported used data from the National Cancer Database [5]. This retrospective analysis of the 3253 high-risk UTUC patients showed that AC was statistically associated with an overall survival benefit. A meta-analysis based on this retrospective analysis showed that AC could improve overall survival, CSS, and disease-free survival, but neoadjuvant chemotherapy was more favorable for UTUC than AC in disease-specific survival [3]. The systematic review and meta-analysis of 24 retrospective analysis studied the efficacy of NAC and AC in UTUC [15]. Across 2 retrospective studies about NAC, NAC improved CSS, with a pooled HR of 0.41 (95%CI 0.22–0.76, *P* = 0.005). Across three cisplatin-based studies about AC, the pooled HR for overall survival was 0.43 (95% CI, 0.21–0.89, *P* = 0.023) compared with those who received RNU alone. For disease-free survival, the pooled HR across two studies of AC was 0.49 (95% CI, 0.24–0.99; *p* = 0.048). Benefit was not seen for non- cisplatin–based regimens in AC. Meta-analysis of 31 retrospective studies with 8100 UTUC patients who underwent perioperative treatments also showed that AC improved overall survival (HR 0.71, 95%CI 0.51–0.89), CSS (HR 0.71, 95%CI 0.54–0.89), and recurrence-free survival (HR 0.49, 95%CI 0.23–0.85) [16]. We adopted propensity score-matching analysis, which can reduce the differences between patient characteristics in each group, and the results were consistent with those of this previous study. Furthermore, we identified the patients who benefitted from AC. We previously reported that patients with serum low sodium or hemoglobin levels have a poor prognosis. The supposed mechanism of these markers may be that cells in UTUC with a poor prognosis may secrete inflammatory cytokines such as interleukin-6 that cause anemia and low serum sodium levels. This preoperative prognostic marker may also be useful in the selection of patients to receive AC. AC did not improve the prognosis of patients with normal sodium and hemoglobin levels because these patients already had a better prognosis with or without AC.

There are several limitations in this study. Although we matched the cohorts by propensity scores, this is the retrospective study. A multi-institutional, prospective, randomized study should be performed to prove the efficacy of AC. In this study, a median of 2 cycles of AC were administered, but the optimal number of cycles was not determined. We entered only serum sodium and hemoglobin levels into the Cox proportional analysis, but other prognostic markers might exist to predict the benefit of AC.

## Conclusion

Propensity score-matched analysis showed that AC improved the survival of patients with advanced UTUC. Subgroups of patients with lower sodium and/or hemoglobin levels could benefit from AC after RNU. Further large-scale studies are required to verify these findings.

## Abbreviations

AC: Adjuvant chemotherapy
HR: Hazard ratio
LVI: Lymphovascular invasion
RNU: Radical nephroureterectomy
UTUC: Upper urinary tract urothelial carcinoma
